# Genomic and transcriptomic insights into complex virus–prokaryote interactions in marine biofilms

**DOI:** 10.1038/s41396-023-01546-2

**Published:** 2023-10-24

**Authors:** Kun Zhou, Tin Yan Wong, Lexin Long, Karthik Anantharaman, Weipeng Zhang, Wai Chuen Wong, Rui Zhang, Pei-Yuan Qian

**Affiliations:** 1 Department of Ocean Science, The Hong Kong University of Science and Technology, Hong Kong, China; 2 Southern Marine Science and Engineering Guangdong Laboratory (Guangzhou), Guangzhou, 511458 China; 3 Department of Bacteriology, University of Wisconsin–Madison, Madison, WI USA; 4 Department of Chemical and Biological Engineering, The Hong Kong University of Science and Technology, Hong Kong, China; 5 Institute for Advanced Study, Shenzhen University, Shenzhen, 518060 China

**Keywords:** Biofilms, Metagenomics, Microbial ecology, Microbiome, Water microbiology

## Abstract

Marine biofilms are complex communities of microorganisms that play a crucial ecological role in oceans. Although prokaryotes are the dominant members of these biofilms, little is known about their interactions with viruses. By analysing publicly available and newly sequenced metagenomic data, we identified 2446 virus–prokaryote connections in 84 marine biofilms. Most of these connections were between the bacteriophages in the *Uroviricota* phylum and the bacteria of *Proteobacteria*, *Cyanobacteria* and *Bacteroidota*. The network of virus–host pairs is complex; a single virus can infect multiple prokaryotic populations or a single prokaryote is susceptible to several viral populations. Analysis of genomes of paired prokaryotes and viruses revealed the presence of 425 putative auxiliary metabolic genes (AMGs), 239 viral genes related to restriction–modification (RM) systems and 38,538 prokaryotic anti-viral defence-related genes involved in 15 defence systems. Transcriptomic evidence from newly established biofilms revealed the expression of viral genes, including AMGs and RM, and prokaryotic defence systems, indicating the active interplay between viruses and prokaryotes. A comparison between biofilms and seawater showed that biofilm prokaryotes have more abundant defence genes than seawater prokaryotes, and the defence gene composition differs between biofilms and the surrounding seawater. Overall, our study unveiled active viruses in natural biofilms and their complex interplay with prokaryotes, which may result in the blooming of defence strategists in biofilms. The detachment of bloomed defence strategists may reduce the infectivity of viruses in seawater and result in the emergence of a novel role of marine biofilms.

## Introduction

Microbial biofilms are surface-attached mixed communities of microorganisms, including eukaryotes (e.g. diatoms and fungi), prokaryotes (bacteria and archaea) and acellular viruses, which are enclosed in a matrix of extracellular polymeric substances (EPSs) [1, 2]. The microbial biofilms are widely distributed on marine substrate surfaces, which include seawater surfaces, coastal rocks, zooplankton, phytoplankton, sea floors, animal bodies and artificial surfaces [2]. They play prominent ecological roles in oceans; specifically, they facilitate the degradation of organic pollutants, contribute to photosynthesis, participate in biogeochemical cycling and influence the productivity of coastal ecosystems [3, 4].

In oceans, prokaryotes dominate marine biofilms [5–7], and the dominant prokaryotes have been thoroughly investigated. Nearly 90 years ago, ZoBell and Anderson showed that biofilm bacteria on bottle glass surfaces outnumbered bacteria in seawater [8]. On abiotic or biotic surfaces, such as marine-grade plywood substrates or macroalgae, prokaryotic density can reach 10^8^ cells per square centimetre [9, 10]. More than 25,000 operational taxonomic units (OTUs) of the 16 S rRNA genes of marine biofilm prokaryotes have been clustered (threshold of sequence identity: 97%) globally [11]. Over 25,000 species are likely to be present in marine biofilms according to an empirical study which demonstrated that 16 S rRNA gene sequence similarity between most strains exceeds 97% [12]. A large number of prokaryotic species constitute more than 30 phyla, such as *Proteobacteria*, *Acidobacteria*, *Actinobacteria* and *Crenarchaeota*, and *Proteobacteria* is the predominant group [6, 11]. Marine biofilms have a distinct microbial community composition, as evidenced by a metagenomic survey that unveiled 7300 OTUs unique to marine biofilms [11]. Despite these findings, prokaryotic interactions with viruses in marine biofilms have not been explored.

Viruses are the most abundant biological entities on Earth [13]. They are a major cause of microbial mortality and help shape the community composition of planktonic prokaryotes [14]. However, viral predation is limited in biofilms, as living in biofilms offers more benefits to microorganisms than seawater, particularly under adverse conditions [15]. In laboratory experiments, biofilm structure and composition were found to inhibit viral predation [7]. For instance, the EPS matrix of a biofilm structure can entrap viruses and inhibit their diffusion, thereby limiting access to prokaryotic cells, such as the cultured bacterium *Pantoea stewartia* [16]. Other mechanisms can be employed by bacteria to counter viral attacks through signalling systems (e.g. quorum sensing) or anti-viral defence systems (e.g. CRISPR–Cas system [CRISPR stands for clustered regularly interspaced short palindromic repeat]) [7]. However, viruses trapped in a biofilm matrix can remain active and infect colonising cells, as demonstrated in T7 phages [17]. One of the ways for viruses to penetrate the EPS matrix and access host cells is to encode depolymerases that degrade polymeric substances [18]. In addition, viruses can use biofilm channels for diffusion to target bacterial hosts [19]. The interplay between bacteria and phages under laboratory conditions indicates complex virus–prokaryote interactions in natural environments and triggers our interest to investigate natural marine biofilms.

In this work, we studied publicly available metagenomic data generated from biofilms and surrounding seawater samples from the South China Sea, East China Sea, Red Sea and South Atlantic [11] and three additional metagenomes of biofilms developed in Hong Kong waters. To demonstrate viral and prokaryotic gene expression, we produced the three metatranscriptomes of biofilms established in Hong Kong waters. Using a large set of omics data, we aimed to predict virus–prokaryote pairs, identify genes related to auxiliary metabolism and counter-defence in viral genomes and determine defence systems in the prokaryotic genomes of biofilms and surrounding seawater. We hypothesise that viruses are active in natural marine biofilms, and the infections they cause lead to the proliferation of prokaryotic defence strategists, which may enhance anti-viral resistance in seawater when they return to a free-living style through detachment.

## Materials and methods

### DNA and RNA extraction and sequencing of biofilms established in Hong Kong waters

Biofilms were developed with polystyrene Petri dishes at Hong Kong waters (22° 20′ 24.0′′ N, 114° 16′ 12.0′′ E, depth of 1–2 m) for gene expression analyses. After a 25-day development period, biofilms were collected in April 2022 and named HK-2022 biofilms. Three biofilm samples on the surfaces were collected immediately with sterile cell scrapers and stored separately in DNA buffer (500 mmol/L NaCl, 50 mmol/L Tris–HCl, 40 mmol/L EDTA and 50 mmol/L glucose referring to [11]) for DNA extraction. The three biofilms were merged into one and immediately transferred to RNAprotect Bacteria Reagent (QIAGEN, Hilden, Germany) for RNA storage. To extract the total DNA of microbiomes including viruses, we used polyethylene glycol (PEG) to concentrate viral particles. In brief, the pH of the virus-containing supernatant (biofilms in DNA buffer) was adjusted to pH 7.5. PEG (MW 6000) was added to a final concentration of 10% (w/v) and incubated at 4 °C for 8 h, followed by centrifugation at 10,000 × *g* for 1 h. Finally, the total DNA of metagenomes was extracted using DNeasy PowerBiofilm kit (QIAGEN, Hilden, Germany) according to the manufacturer’s protocol. Libraries with an insert size of approximately 350 bp were constructed and sequenced on the HiSeq X Ten platform (Illumina) with a read length of 150 bp (Novogene, Beijing, China). For RNA extraction, the total RNA of merged biofilms was extracted using the Rneasy PowerBiofilm kit (Qiagen, Hilden, Germany) according to the manufacturer’s protocol. Total RNA was divided into three repeats, and they were separately sequenced using the NovaSeq platform (Illumina) by Novogene Company (Beijing, China) with a 150 bp short-insert library to generate 10 Gb paired-end reads for each repeat sample.

### Metagenome assembly and identification of viral sequences

**Fig. 1:**
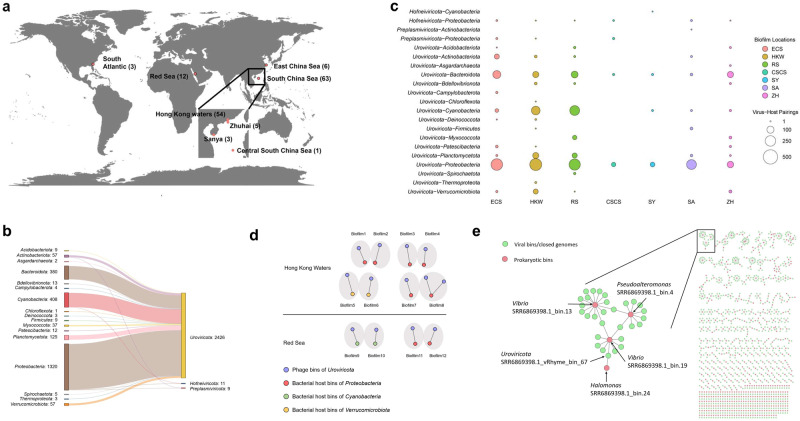
Virus–host pairs and their distribution in marine biofilms. **a** Global location of marine biofilms in the study. The quantities of datasets are indicated by numbers enclosed in brackets. The ocean map underwent modification using ArcGIS online maps (available at https://www.arcgis.com/). **b** Virus–prokaryote connections at the phylum rank. The number of pairs was indicated after each phylum. **c** Distribution of virus–prokaryote pairs in biofilms in the oceans. ECS: East China Sea (30° 42′ 00.0′′ N 122° 49′ 12.0′′ E). HKW: Hong Kong waters (22° 20′ 24.0′′ N 114° 16′ 12.0′′ E). RS: Red Sea (22° 12′ 00.0′′ N 39° 01′ 48.0′′ E). CSCS: Central South China Sea (14° 00′ 00.0′′ N 116° 00′ 00.0′′ E). SY: Sanya (18° 13′ 48.0′′ N 109° 29′ 24.0′′ E). SA: South Atlantic (31° 25′ 12.0′′ N 81° 18′ 00.0′′ W). ZH: Zhuhai (21° 42′ 00.0′′ N 114° 21′ 00.0′′ E). **d** Shared phage–bacteria pairs in different biofilms from Hong Kong waters (e.g. Biofilm1 shared *Uroviricota*–*Proteobacteria* pairs with Biofilm2) and the Red Sea (e.g. Biofilm9 shared *Uroviricota*–*Cyanobacteria* pairs with Biofilm10). Biofilm1: HK-2022-1. Biofilm2: HK-2022-2. Biofilm3: SRR6854594.1. Biofilm4: SRR6854592.1. Biofilm5: SRR6854597.1 Biofilm6: SRR6854601.1. Biofilm7: SRR6854599.1. Biofilm8: SRR6854598.1. Biofilm9: SRR6869052.1. Biofilm10: SRR6869055.1. Biofilm11: SRR6869053.1. Biofilm12: SRR6869051.1. **e** Complex virus–prokaryote pairs in marine biofilms. A subnetwork highlighted within a black box was magnified on the left side to provide detailed information.

The sequenced reads of three HK-2022 biofilms and the raw Illumina reads of publicly available biofilms and seawater samples (Fig. 1a and Table S1) from a previous study [11] were retrieved from the NCBI database (BioProject accession: PRJNA438384). Raw reads were trimmed by Trimmomatic v0.36 [20] with custom parameters (ILLUMINACLIP: TruSeq3-PE.fa:2:30:10 LEADING:3 TRAILING:3 SLIDINGWINDOW:4:15 MINLEN:40) to remove adaptors and low-quality reads. The quality-controlled reads were assembled using SPAdes v3.11.1 (--meta) [21]. Viral sequence candidates (scaffolds ≥5 kb) were identified from assembled metagenomic sequences by multiple identifiers, including VirSorter v1.0.5 (searching against the RefSeqABVir database; hallmark gene number ≥1) [22], VirSorter2 v2.2.3 (score≥0.9 and/or hallmark gene number ≥1) [23], VIBRANT v1.2.0 (categorised as lytic or lysogenic phages) [24], Seeker (score≥0.7) [25] and DeepVirFinder (score≥0.9, *p* < 0.05) [26], which rely on protein similarity and/or machine-learning models. Given that eukaryotic scaffolds might be misidentified as viral sequences by DeepVirFinder or Seeker, eukaryotic sequences were recognised by CAT v4.6 (--fraction 1 at phylum rank) against the NCBI-nr database [27]. When a viral sequence candidate belonged to the identified eukaryotes, this sequence candidate was removed. The remaining scaffolds, classified as low-quality, medium-quality, high-quality or complete sequences by CheckV v0.7.0 (end_to_end) [28], were identified as viral sequences.

### Identification of proviral sequences and closed viral genomes

Some putative viral scaffolds might be derived from proviruses that are components of host chromosomes. The proviral scaffolds were predicted based on the following criteria: (1) scaffolds were classified as sequences containing proviruses by CheckV v0.7.0 (end_to_end) [28]; and (2) scaffolds were from the prokaryotic bins that were extracted using metaWRAP v1.2 (-l 1000 bp -metabat2 -maxbin2 -concoct; bin_refinement; completeness ≥50, contamination ≤10) [29]. Referring to a prior study [28], CheckV was used to predict closed viral genomes that should meet all the following criteria: (1) scaffolds were in the type of DTR; (2) scaffolds were not obtained from proviruses identified above; (3) scaffolds did not contain low-complexity repeats; (4) repeats were without Ns that represented gaps; (5) repeat number should be lower than six in one scaffold; and (6) the repeat region must be less than 20% of the scaffold in length.

### Binning of viral genome fragments

In the binning of scaffolds, proviral and closed sequences identified above were removed. The remaining scaffolds were individually clustered in each sample with vRhyme v1.0.0 (default parameters) on the basis of read coverage and sequence composition [30]. Viral bins were further filtered to remove the bins of mixed populations (one bin consisting of different populations). Genome bins were firstly imported into Prodigal v2.6.3 (-m -p meta) [31] for gene prediction. In the following, the predicted genes were aligned to the viral RefSeq database from NCBI (https://www.ncbi.nlm.nih.gov) and individually classified using a Last Common Ancestor algorithm [32] embedded in the Contig Annotation Tool (CAT) v4.6 [27]. A majority rule (--fraction 0.5), where >50% of the sum of bit-scores of all genes supports the classification, was employed to assign taxonomy at the phylum level to each sequence. Given that CAT has not been tested on viral genomes, it was tested using viral genomes derived from GenBank (https://www.ncbi.nlm.nih.gov/) before employment. The results showed that CAT could generate classification that was largely consistent with the International Committee on Taxonomy of Viruses at the phylum level (3804 out of 3805 GenBank genomes consistent; Table S2). In addition, CAT was tested using GenBank viral genomes in *Duplornaviricota*, *Dividoviricota*, *Hofneiviricota*, *Phixviricota*, *Preplasmiviricota* and *Uroviricota*, which were the outputs of annotation on vRhyme bins from CAT. Similarly, testing results on the six phyla showed high congruence (> 96%) with NCBI taxonomy (Table S3). Additionally, HMMScan in HMMER v3.3 tool suite [33] with parameters (--notextw -E 1e-5; bit score ≥30) was used to search terminase large subunit (TerL) genes against Pfam v35.0 [34]. Finally, when a bin contained sequences from more than one phylum or the bin displayed multiple TerL genes, the bin was removed because it may belong to different populations. The removed scaffolds were added to the pool of genomic fragments.

### Prediction of virus–prokaryote connections

The identified viral genome fragments, genome bins and complete genomes were classified at the phylum level using the Last Common Ancestor algorithm and the majority rule. Viral RefSeq viruses were searched as mentioned above. Sequences or bins were selected as prokaryotic virus candidates when they contained viral genes identified by CheckV and were associated with *Dividoviricota*, *Duplornaviricota*, *Hofneiviricota*, *Phixviricota*, *Preplasmiviricota* and *Uroviricota*, which infect prokaryotes according to the Virus–Host DB (https://www.genome.jp/virushostdb/) [35]. Whether the candidates are viruses infecting prokaryotes was determined by virus–host prediction. Putative virus–host connections between the virus candidates and prokaryotic bins extracted with metaWRAP from biofilm microbiomes were identified according to any of the following criteria: (1) sequences from a viral fragment/bin/complete genome and scaffolds from a prokaryotic bin had ≥70% BLASTn identity (E-value ≤ 10^−3^) and ≥2.5 kb alignment length [36, 37]. (2) The CRISPR spacers >6 bp predicted with MetaCRT [38, 39] from a prokaryotic genome bin identically matched the genome sequences of a viral fragment/bin/complete genome [37, 40] with fuzznuc [41]. (3) The tRNA genes from a viral fragment/bin/complete genome were identical to the tRNA from a prokaryotic bin (using BLASTn) [40, 42]. (4) Sequences from a viral fragment/bin/complete genome and scaffold(s) from a prokaryotic bin shared exact matches (*k*-mer length = 25) after alignment-free PHIST v1.0.0 with default parameters was used [43].

### Identification of shared virus–prokaryote pairs between biofilms

Viral and prokaryotic genomes were assigned to ‘populations’ based on average nucleotide identity (ANI). FastANI v.1.33 was employed to calculate ANI for viruses with custom parameters (--fragLen 500 -minFraction 0.8) and for prokaryotic hosts with a custom setting (-minFraction 0.5) [44]. If the paired viruses and prokaryotes in different biofilms belonged to the same populations (ANI ≥ 95%), then the virus–prokaryote pairs were shared between the compared biofilms.

### Calculation of average read coverage and virus-to-prokaryote ratios

Paired viral and prokaryotic genome sequences, along with metagenomic reads, were input into Bowtie2 version 2.3.4 [45] and SAMtools version 1.6 [46] to calculate the average sequencing depth with default parameters. Viral bins and closed genomes that can represent viral populations and their prokaryotic host genomes were selected to estimate virus-to-prokaryote ratios. A prokaryotic host paired with multiple viruses indicated the accumulation of the read coverage of all the viruses.

### Gene calling and taxonomic annotation of prokaryotic hosts

In the gene prediction of prokaryotes, the open reading frames (ORFs) of genomes of bacteria and archaea were predicted by performing Prodigal [31] with customised settings (-c, -m). For the taxonomic annotation of bacterial and archaeal bins, the predicted genes were fed into GTDB-Tk v0.3.1 [47], using the ‘classify_wf’ parameter, to identify single-copy marker genes that were then analysed for prokaryotic classification with GTDB taxonomy [47].

### Identification of auxiliary metabolic genes

Prodigal [31] with customised settings (-c, -m) was used to analyse genomes of viruses paired with prokaryotes to predict ORFs. Subsequently, the ORFs were searched against the Kyoto Encyclopedia of Genes and Genomes (KEGG) database [48] using KofamScan version 1.2.0 (E-value < 10^−5^, score>predefined thresholds by KofamScan) [49]. The ORFs were imported into HMMScan (E-value < 10^−3^ and bit score>30) [50] for further annotation based on the PFAM database [34]. ORFs with KEGG and PFAM annotation were then searched against a viral AMG database derived from previous studies [37, 51–58] including experimentally verified AMGs [51, 53–55, 57], and a set of PFAM and KEGG accessions of the AMGs was retrieved. ORFs with the retrieved PFAM and KEGG accessions were retained and incorporated into the set of AMGs. After identification based on customised scripts, VIBRANT v1.2.0 was used to automatically predict other possible AMGs, which were classified into the category of KEGG metabolic pathways. Lastly, gene position in viral scaffolds (in the classifications of genome fragments and bins) and functional annotation of all the putative AMGs were manually checked.

### Identification of defence and counter-defence genes

Prokaryotic defence system-related gene candidates were identified by searching against Prokaryotic Antiviral Defence System (PADS) [59] using the DIAMOND BLASTp command (more sensitive mode, identity ≥30%, E-value < 10^−10^) [60]. To verify the presence of conserved domains of the antiphage defence gene, we annotated the identified gene candidates using HMMScan in HMMER 3.3 tool suite [33] against PFAM 32.0 [34] (E-value < 10^−3^, bit score≥30). The PFAM accessions of the conserved domains of antiphage defence genes [61] were used to check their presence in the annotated gene candidates. Gene sequences containing the conserved domains were retained and incorporated into the set of defence-related genes of prokaryotes. Similar processes used to predict prokaryotic defence genes were performed to identify counter-defence genes in viruses. Viral genomes were imported into DIAMOND BLASTp and HMMScan in HMMER to search against PADS and PFAM, respectively. Viral genes containing the conserved domains of RM system-related genes were considered counter-defence genes. We detected the gene components of a system in a contig sequence or a bacterial bin as previously described to predict the completeness of defence systems [62–66]. The system was considered complete when it included all the genes required.

### Transcriptome assembly and gene expression quantification for the microbiomes of HK-2022 biofilms

Sequenced raw RNA reads of the metatranscriptome of three biofilm repeats were trimmed by Trimmomatic (version 0.36) with custom parameters (ILLUMINACLIP: TruSeq3-PE.fa:2:30:10 LEADING:3 TRAILING:3 SLIDINGWINDOW:4:15 MINLEN:40) to remove Illumina adapters and low-quality bases of RNA reads. The trimmed reads were processed with Trinity v2.8.5 with default parameters [67] to de novo assemble metatranscriptomes. Reconstructed transcripts were mapped to the predicted genes of metagenomes of viruses/prokaryotes of the HK-2022 biofilms using BLASTn (E-value < 10^−3^, identity ≥95%, coverage = 100%). Simultaneously, Salmon with default parameters and an input of RNA-sequencing reads [68] was used in the quantification in transcripts per million (TPM) to assess expression levels of viral/prokaryotic genes. In this study, we defined the expression of a viral/prokaryotic gene as having the support of at least one transcript from one repeat sample or read mapping to all repeat samples.

### Abundance of anti-viral defence genes in prokaryotes of biofilms and seawater

We used available metagenome samples from Hong Kong waters (biofilm established on polystyrene panels: *n* = 55, seawater: *n* = 11) and the Red Sea (biofilm established on zinc panels: *n* = 12, seawater: *n* = 12) to compare the abundance of defence-related genes between prokaryotes in biofilms and their ambient seawater. Metagenomic sequences classified as prokaryotes by CAT and sequences of metagenome-assembled bins were selected for the following analyses. Referring to the above-mentioned section *Identification of defence and counter-defence genes*, anti-viral defence genes were identified. According to the pipeline proposed by Jin Choi (https://github.com/edamame-course/Metagenome/blob/master/2016-07-15-counting-abundance-with-mapped-reads.md), metagenomic reads were mapped to the identified defence genes with Bowtie2 version 2.3.4 [45], and aligned reads were counted using SAMtools version 1.6 [46]. RPKM was calculated for each sample to estimate gene abundance. The relative abundance of defence-related genes was calculated by dividing the RPKM of one gene by the sum of RPKM for each location and sample type. DESeq2 1.38.3 package [69] was employed to calculate the differential abundance of defence-related genes between paired sample types from each location. Then, the significantly differentially abundant genes (adjusted *p*  <  0.05) were recognised with a meta-analysis random effects model embedded in R package metafor 3.8-1 [70], to which the log2-fold change value and its associated standard error were input.

## Results

### Virus–prokaryote pairs and distribution

The identification of viral sequences from metagenomes resulted in the generation of three genome datasets. These datasets comprised closed genomes, which were regarded as potential complete genomes; genome bins, encompassing groups of fragmented genomes; and genome fragments, representing unbinned viral scaffolds. The three genome datasets were utilised for pairing with prokaryotic genome bins. A total of 2446 connections (Fig. 1b) were identified between prokaryotes (902 genome bins) and viruses (102 closed genomes, 884 viral bins and 1155 viral genome fragments) from 84 marine biofilms (Supplementary Tables S4–S6). For the prokaryotes with connections, 17 phyla were identified: two archaeal phyla (*Asgardarchaeota* and *Thermoproteota*) and 15 bacterial phyla (*Acidobacteriota*, *Actinobacteriota*, *Bacteroidota*, *Bdellovibrionota*, *Campylobacterota*, *Chloroflexota*, *Cyanobacteria*, *Deinococcota*, *Firmicutes*, *Myxococcota*, *Patescibacteria*, *Planctomycetota*, *Proteobacteria*, *Spirochaetota* and *Verrucomicrobiota*). For the prokaryotic viruses in connections, the phyla *Hofneiviricota*, *Preplasmiviricota* and *Uroviricota* were assigned to viruses. Most of the identified connections were between bacteriophages in the phylum of *Uroviricota* and the bacteria of *Proteobacteria*, *Cyanobacteria* and *Bacteroidota*.

The viruses and prokaryotes constituted 21 phylum–phylum pairs and were widely distributed in oceans (Fig. 1c). The *Uroviricota*–*Proteobacteria* and *Uroviricota*–*Bacteroidota* pairs were found in most of the biofilms of the South China Sea, East China Sea, Red Sea and South Atlantic, indicating a wide distribution in oceans. By contrast, the *Uroviricota*–*Thermoproteota* pair was specific to biofilms of Hong Kong waters. In addition to the pairing between *Uroviricota* and *Thermoproteota*, many other virus–prokaryote pairs, such as *Hofneiviricota* and *Cyanobacteria*, were exclusively found in a specific location with a small number of biofilms, suggesting that the distribution of viruses and their prokaryotic hosts displayed an endemic feature in marine biofilms. Moreover, the analysis of the average nucleotide identity (ANI; ≥95%) of closed/binned viral genomes and prokaryotic genome bins showed the absence of shared virus–host pairs in different environments. By contrast, in a specific environment, such as the Red Sea or Hong Kong waters, viral and host populations were shared among biofilms (Fig. 1d and Supplementary Table S7). In the biofilms from the Red Sea, two populations of *Uroviricota*–*Proteobacteria* and *Uroviricota*–*Cyanobacteria* pairs were shared among different biofilms. In Hong Kong waters, nine shared pairs (*Uroviricota*–*Proteobacteria* and *Uroviricota*–*Verrucomicrobiota*) were identified. The viral and host populations of *Uroviricota* and *Proteobacteria*, respectively, were present in biofilm3 (SRR6854594.1_vRhyme_bin_3 and SRR6854594.1_bin.2) and biofilm4 (SRR6854592.1_vRhyme_bin_4 and SRR6854592.1_bin.2).

The clustering of paired viral and prokaryotic genomes generated 433 groups, of which 184 were composed of more than one viral or prokaryotic genome (Fig. 1e). The virus–host pair network reflected a complex relationship between viruses and prokaryotes in marine biofilms. Such a relationship indicated that a single virus could infect multiple prokaryotic populations or a single prokaryote is susceptible to several viral populations. For instance, *Vibrio* SRR6869398.1_bin.13 was paired with 14 viral bins, such as SRR6869398.1_vRhyme_bin_100 in the phylum of *Uroviricota*, and *Uroviricota* SRR6869398.1_vRhyme_bin_67 was related to *Halomonas* SRR6869398.1_bin.24 and *Vibrio* SRR6869398.1_bin.19 (Fig. 1e). The infection of a single prokaryote by several viral populations might lead to high virus-to-prokaryote ratios. According to the analysis of average metagenomic read coverage, many phage–bacterium pairs had high phage-to-bacterium ratios (Supplementary Table S8). A total of 52 bacterial bins had ratios of over 10, which were distributed in biofilms sampled from the South China Sea, East China Sea, Red Sea and South Atlantic. Notably, the ratios of phage to bacterium in the biofilms derived from the South Atlantic reached up to 645. The bacteria with such high ratios were affiliated with *Proteobacteria*, *Firmicutes* and *Planctomycetota*, and their paired phages were affiliated with *Uroviricota*.

### Diverse auxiliary metabolic genes in viral genomes paired with prokaryotes

**Fig. 2:**
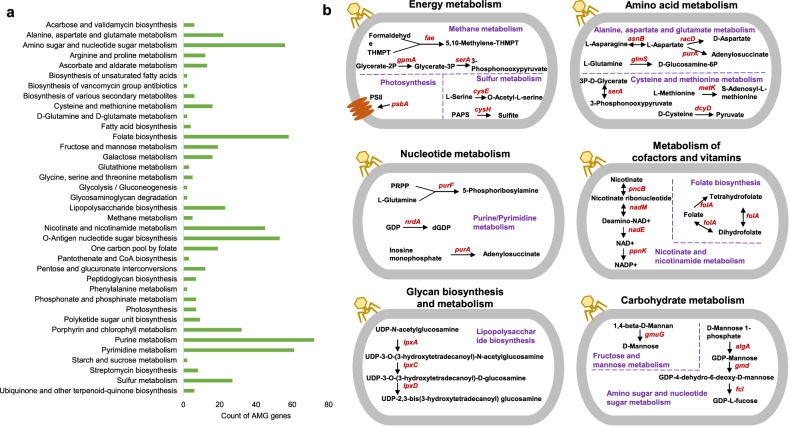
Auxiliary metabolic genes of viruses paired with prokaryotes from 84 marine biofilms. **a** Count of AMGs in each pathway. Here, only pathways containing gene count>1 are shown. **b** Schematic of representative AMG-involved pathways with relatively abundant genes. Pathways were highlighted in violet. AMGs were highlighted in dark red.

Analysis of viral genomes paired with prokaryotic genomes predicted 425 auxiliary metabolic genes (AMGs; Supplementary Table S9). These putative metabolic genes were related to 57 pathways, of which 37 contained more than one metabolic gene (Fig. 2a). Most of the identified AMGs were classified into the pathways of purine metabolism (72 genes), pyrimidine metabolism (61 genes), folate biosynthesis (58 genes), amino sugar and nucleotide sugar metabolism (56 genes), O-antigen nucleotide sugar biosynthesis (53 genes), and nicotinate and nicotinamide metabolism (45 genes). A total of 61 and 7 genes were the homologues of the genes *nrdA* and *psbA*, respectively, which are two experimentally verified genes that play a crucial role in viral replication [51, 53–55, 57]. The gene *nrdA* [57] encodes the ribonucleoside–diphosphate reductase alpha chain that converts guanosine diphosphate into deoxyguanosine diphosphate (Fig. 2b), whereas the gene *psbA* codes for the photosystem II P680 reaction centre D1 protein [51, 53–55], which is involved in photosynthesis. Genes homologous to *nrdA* occurred in almost all the biofilm locations of the Red Sea, South China Sea, East China Sea and South Atlantic. The temporal and spatial distributions of *nrdA* reflected the stable presence of nucleotide metabolism-related AMGs in marine biofilms (Figs. S3 and S4). On the basis of taxonomic annotation, *nrdA* and *psbA* were mainly from the *Uroviricota* viruses, which infect *Actinobacteriota*, *Bacillota*, *Bacteroidota*, *Cyanobacteria*, *Planctomycetota*, *Proteobacteria* and *Verrucomicrobiota*. *Proteobacteria* and *Cyanobacteria* accounted for a large proportion.

### Counter-defence and anti-viral genes in paired viral and prokaryotic genomes

In the arms race between viruses and microbes, bacteria can evolve innate and adaptive immunity, including systems for restriction–modification (RM) [71], defence island system associated with restriction–modification (DISARM) [72], bacteriophage exclusion (BREX) [73] and CRISPR–Cas [74], to target invading DNA for defence. They can develop toxin–antitoxin (TA) [75] and abortive infection (ABI) [76] to abort viral replication and initiate programmed death. Systems with unknown mechanisms, such as the Zorya, Hachiman, Gabija, Septu, Thoeris, Lamassu, Druantia, Wadjet, Kiwa and Shedu, have evolved in some bacteria [61]. Conversely, viruses have the capability to undergo mutations as a means of evading these defences, thereby enhancing their fitness. One well-established counter-defence mechanism involves the development of RM systems to withstand host defence by employing anti-restriction strategies [77].

**Fig. 3:**
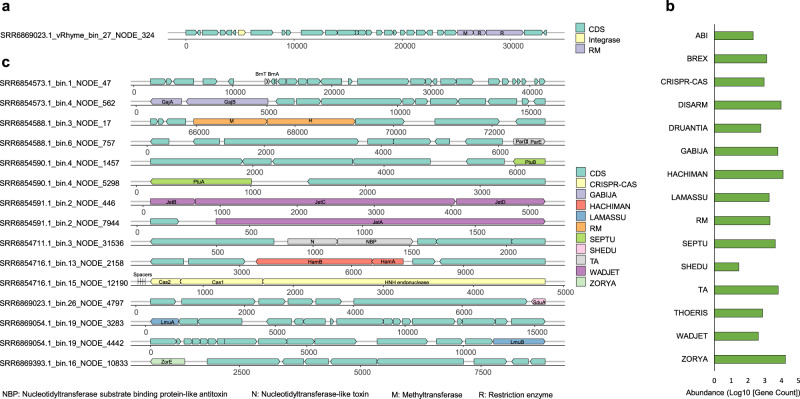
Defence system-related genes in paired viral and prokaryotic genomes from 84 marine biofilms. **a** Schematic of gene composition of a representative RM system in a temperate viral genome bin. SRR6869023.1_vRhyme_bin_27 represents a virus in the phylum of *Uroviricota* from the biofilm established on rocks situated in Hong Kong waters. **b** Count of genes in each system. The horizontal axis represents the value of Log10 [Gene Count]. **c** Anti-viral defence systems in marine biofilm prokaryotes with a complete set of required system components. Bacteria of biofilms developed on Petri dishes at Hong Kong waters: *Cyanobacteria* (SRR6854573.1_bin.1, SRR6854590.1_bin.4 and SRR6854711.1_bin.3), Verrucomicrobiota (SRR6854573.1_bin.4) and *Proteobacteria* (SRR6854588.1_bin.3, SRR6854588.1_bin.6, SRR6854591.1_bin.2, SRR6854716.1_bin.13 and SRR6854716.1_bin.15). SRR6869023.1_bin.26 represents a bacterium in the order of *Cyanobacteria* established on rocks at Hong Kong waters. SRR6869054.1_bin.19 is in the order of *Proteobacteria* from the biofilm developed on zinc panels at the Red Sea. SRR6869393.1_bin.16 represents a bacterium of *Proteobacteria* from the biofilm developed on Petri dishes at the East China Sea. Green represents genes encoding non-defence or unknown functions.

We detected 239 viral genes related to RM systems encompassing types I, II and III (details in Supplementary Table S10). Biofilm viruses encoding RM systems were distributed widely from the East China Sea to the South China Sea and Red Sea. Specifically, they were identified in biofilms that grew on the beaches and rocks of Hong Kong; zinc panels at the Red Sea; and polystyrene dishes in the East China Sea, South China Sea and South Atlantic. For example, three genes that are near an integrase gene and encode N-6 DNA methylase, type I RM DNA specificity domain and type III restriction enzyme in the viral bin SRR6869023.1_vRhyme_bin_27 were present in the rock biofilm at Hong Kong waters (Fig. 3a). Type II RM systems including genes encoding type II restriction endonuclease were detected in SRR6869046.1_vRhyme_bin_18 from the Red Sea biofilms.

A total of 38,538 anti-viral defence-related genes were detected in 902 genome bins of biofilm prokaryotes paired with viruses (Table S11). These genes were involved in 15 defence systems encompassing Zorya, Hachiman, defence island system associated with RM (DISARM), TA, Gabija, RM, Septu, Lamassu, Brex, CRISPR–Cas, Thoeris, Druantia, Wadjet, abortive infection (ABI) and Shedu (Fig. 3b). Amongst these systems, the Zorya, Hachiman, DISARM, TA, Gabija and RM displayed a large number of genes, comprising the main gene components. In terms of completeness of systems, Zorya, Hachiman, Lamassu, Gabija, Wadjet, Shedu, Septu, TA, RM and CRISPR–Cas systems showed a full set of required defence genes (Fig. 3c; details in Supplementary Results).

### Defence gene composition differs in biofilms and surrounding seawater

**Fig. 4:**
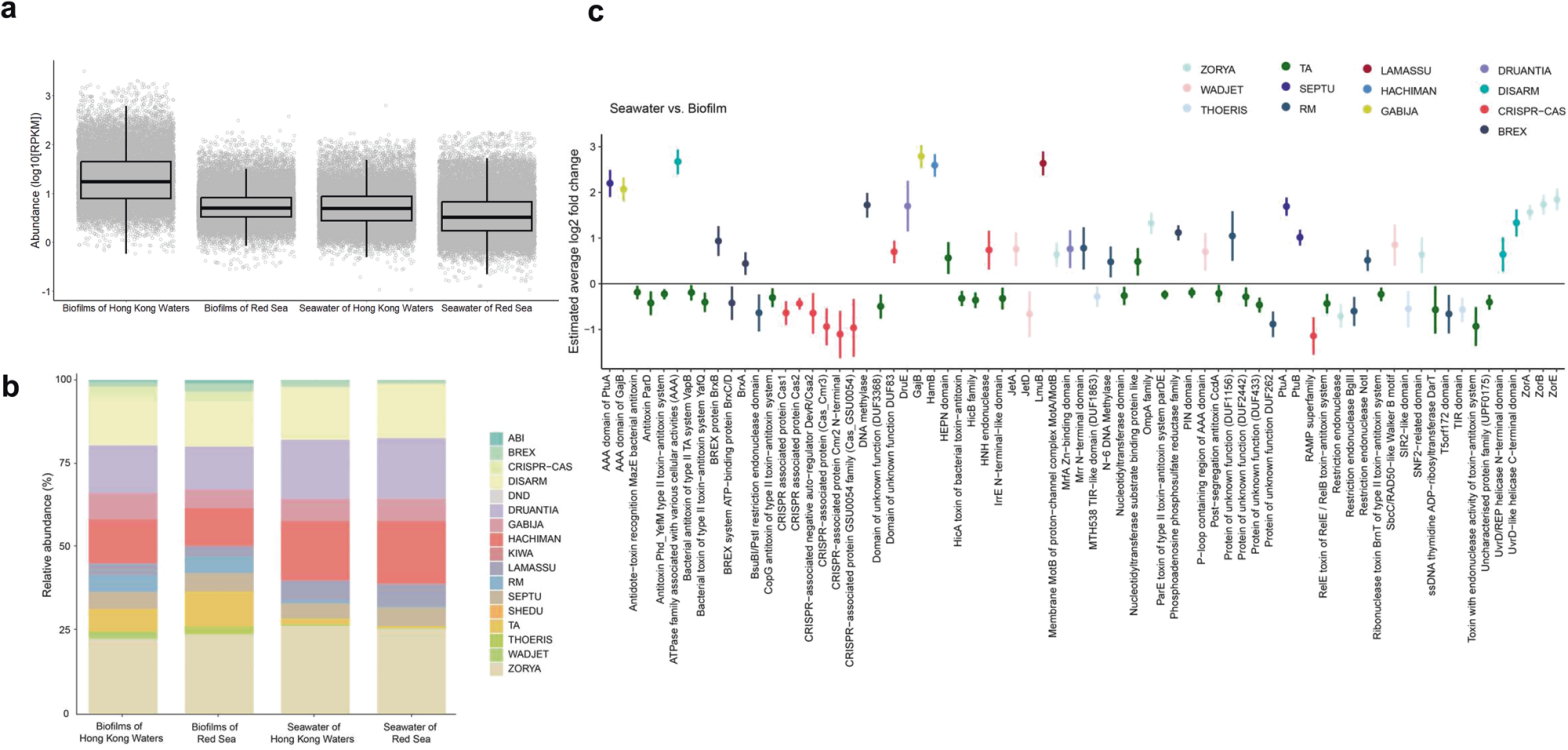
Comparison of defence-related gene abundance between biofilms and surrounding seawater from Hong Kong waters and Red Sea. **a** Absolute abundance in log10 of reads per kilobase of read per million (RPKM) of defence-related genes for paired samples of biofilms and seawater from Hong Kong waters (biofilm established on polystyrene panels: *n* = 55, seawater: *n* = 11) and the Red Sea (biofilm established on zinc panels: *n* = 12, seawater: *n* = 12). The horizontal line that splits the box is the median, the upper and lower sides of the box are upper and lower quartiles, whiskers are 1.5 times the interquartile ranges and data points beyond whiskers are considered potential outliers. **b** Relative abundance of reads labelled by defence systems across all samples from Hong Kong waters (biofilm established on polystyrene panels: *n* = 55, seawater: *n* = 11) and the Red Sea (biofilm established on zinc panels: *n* = 12, seawater: *n* = 12). **c** Estimated average log2 fold change of defence-related genes between paired biofilm and seawater samples from Hong Kong waters and the Red Sea via random effects meta-analysis (*p*  <  0.05). Error bars represent 95% confidence intervals. Defence genes (adjusted *p*  <  0.05 from differential abundance analysis) selected for the meta-analysis between paired samples of biofilms and seawater from Hong Kong waters (biofilm established on polystyrene panels: *n* = 55, seawater: *n* = 11) and the Red Sea (biofilm established on zinc panels: *n* = 12, seawater: *n* = 12).

Here, we regarded all the prokaryotic defence genes as anti-viral defensomes. To investigate the differences between biofilm and seawater defensome profiles, we identified the defence genes in prokaryotic communities from Hong Kong waters and the Red Sea with sufficient samples. We then compared the abundance, measured as reads per kilobase of gene per million mapped reads (RPKM), between biofilm and seawater samples. In total, 16,563 and 18,996 genes were separately identified in biofilm and seawater samples of Hong Kong waters, and 24,043 and 21,498 genes were identified in the biofilm and seawater samples of the Red Sea, respectively (Tables S12–15). The total abundance of defence-related genes in biofilms developed on polystyrene Petri dishes at Hong Kong waters was higher than that in other samples, including the seawater samples from Hong Kong waters and the biofilms developed on zinc panels and seawater of the Red Sea (Fig. 4a). Biofilms developed on zinc panels at the Red Sea and seawater of Hong Kong waters had a slightly higher abundance than seawater samples from the Red Sea (Fig. 4a). Although the biofilms of Hong Kong waters had a higher abundance of defence genes than the biofilms of the Red Sea, they had similar system compositions (Fig. 4b and Table S16). Additionally, the seawater of Hong Kong and the Red Sea had similar defence system compositions. Biofilm samples from both Hong Kong waters and the Red Sea contained a higher relative abundance of defence-related genes coding for the systems of ABI, CRISPR–Cas, Kiwa, RM, Shedu, TA, Thoeris and Wadjet than seawater samples (Fig. 4b). By contrast, defence-related genes coding for the systems of DISARM, Druantia, Hachiman, Lamassu and Zorya were higher in abundance in seawater prokaryotes than in biofilm samples.

The comparison amongst the abundance levels of the defence-related genes showed that seawater samples were enriched with 33 genes coding for 13 defence systems (Fig. 4c). Defence genes encoding LmuB of Lamassu, ATPase family associated with various cellular activities (AAA) of DISARM and GajB of GABIJA in seawater prokaryotes had the highest log fold changes (2.79, 2.67 and 2.63, respectively), whereas genes for BrxA of BREX, N-6 DNA methylase of RM and nucleotidyltransferase substrate binding protein-like antitoxin of TA had the lowest log fold changes (0.44, 0.47 and 0.48, respectively) compared with those of the biofilm samples (Fig. 4c). Biofilm samples were enriched with 38 genes coding for six systems (CRISPR–Cas, TA, RM, Wadjet, Thoeris and BREX; Fig. 4c). The highest log fold changes were observed in genes encoding the RAMP superfamily, Cmr2, GSU0054 family and Cmr3 of CRISPR–Cas from biofilms ( − 1.15, −1.11, −0.97 and −0.94, respectively) compared with seawater samples. By contrast, genes for type II TA systems encompassing bacterial antitoxin VapB, the PIN domain and antitoxin MazE had the lowest log fold changes (− 0.196, −0.195 and −0.192, respectively).

### Expression of anti-viral defence genes, hallmark genes, auxiliary metabolic genes and counter-defence genes in the microbiomes of HK-2022 biofilms

For the prokaryotes paired with identified viruses in biofilm microbial communities, sequenced read and reconstructed transcript mapping showed that genes related to all the detected anti-viral systems of Zorya, Hachiman, DISARM, TA, Gabija, RM, Septu, Lamassu, Brex, CRISPR–Cas, Thoeris, Druantia, Wadjet and Shedu were transcribed by *Actinobacteriota*, *Bacteroidota*, *Planctomycetota* and *Proteobacteria* (Table S17). Some defence systems had a high level of gene expression, such as the type II-C CRISPR–Cas in *Alteromonadaceae* biofilm1_bin.9 targeting phages in *Uroviricota* (Table S17). The TPM values of genes for Cas2, Cas1 and HNH endonuclease in type II-C CRISPR–Cas were high compared with those of other defence genes. In particular, the TPM of the Cas1 gene for spacer insertion could reach up to 604.

Transcriptomic analysis also showed that the genes of viruses paired with prokaryotic hosts were expressed (Supplementary Tables S18–20 and Supplementary Results). Transcriptomic read and transcript mapping supported the expression levels of 35 AMGs, which were involved in 13 pathways: folate biosynthesis; sulphur metabolism; purine and pyrimidine metabolism; pentose phosphate pathway; glycosaminoglycan degradation; methane metabolism; photosynthesis; porphyrin and chlorophyll metabolism; glutathione metabolism; cysteine and methionine metabolism; glycine, serine and threonine metabolism; and one-carbon metabolism (Table S19). Amongst these AMGs, the gene *nrdA* associated with purine and pyrimidine metabolisms accounted for a large proportion (15 genes). An assessment of expression level showed that the viral *nrdA* was highly expressed in biofilms. The TPM of *nrdA* in the unbinned scaffold NODE_184 of biofilm1 ranged from 8.5 to 16.3, whereas the TPM of all the other expressed AMGs in biofilm1 was below 8.5 (Table S19). As for viral genes related to counter-defence, though few genes had read support, the type III restriction enzyme genes were active in three samples, indicating the expression of the counter-defence systems of the viruses.

## Discussion

Coastal marine environments are characterised by their unforgiving conditions, which include temperature fluctuations, pH variations, wave action, evaporation and salinity changes [78]. These challenging environmental factors compel prokaryotes to adopt a sessile lifestyle by attaching to natural and man-made surfaces. Remarkably, this strategy has been adopted by cellular organisms for billions of years, as evidenced by fossil records [79, 80]. Marine biofilms, formed as a result of this attachment strategy, serve as protective shields for individual cells against various types of environmental stressors, including the threat of predation by other organisms [81]. This protective environment fosters the development of a diverse microbial community and enables these microorganisms to thrive within marine biofilms.

Where there is life, viruses are present [13]. Our study has provided evidence for the presence of viruses in marine biofilms worldwide. These viruses infect a significant proportion of prokaryotes within marine biofilms, as indicated by the relative abundance analysis of metagenomic reads, which revealed virus-prokaryote pairings in the majority of prokaryotic genome bins across the various marine biofilms (detailed results in Figure S1). Our results unveiled 2446 virus–prokaryote pairs in marine biofilms developed at eight locations from the South China Sea, East China Sea, Red Sea and South Atlantic. The connected viruses and their hosts included three phyla of viruses and 17 phyla of bacteria and archaea. The highly diverse and widespread connections suggested interactions between viruses and prokaryotes in marine biofilms. Additionally, we detected the expression of genes in viral sequences from HK-2022 biofilms, including the viral sequence biofilm1_NODE_68 with a high virus–host ratio (about 10:1) that has expressed genes encoding phage tail tube proteins (Table S20). This evidence demonstrates that viruses are active to infect hosts in natural marine biofilms.

Viruses can employ diverse mechanisms to aid in their infections. One such mechanism involves exploiting host metabolisms for replication. Notably, genes related to nucleotide mechanism, such as nrdA, are frequently observed and highly expressed in biofilms. This result suggests that viruses in marine biofilms have evolved efficient strategies to produce purine and pyrimidine for DNA replication. Moreover, energy metabolism plays a pivotal role in viral replication. During infection, viruses harness the energy generated by the host’s metabolism [82]. For instance, in the case of phage T4, viral infection consumes nearly double the host’s normal energy supply, with a burst size of 1000 [83]. In our study, we detected gene homologues of *psbA* in marine biofilm viruses. Viruses carrying *psbA* genes may prevent photo-inhibition in infected cells, ensuring the continuity of photosynthesis and supplying the necessary energy for viral replication [55]. Beyond *psbA* and *nrdA*, we identified hundreds of additional auxiliary metabolic genes. These metabolic genes are associated with over 60 pathways, encompassing amino acid metabolism, nucleotide metabolism, energy metabolism and fatty acid biosynthesis. Thus, a complex interplay exists between viruses and host metabolisms, potentially facilitating viral replication and representing evolutionarily conserved and essential mechanisms for nutrient digestion and energy generation by the host [82].

In addition to employing AMGs, viruses can develop counter-defence systems to enhance their ability to infect host organisms. During infection, viruses likely encounter restriction enzymes derived from the hosts’ RM defence systems, which are detected in 90% of bacterial and archaeal genomes [84]. In phages, foreign DNA-targeting RM systems have evolved as counter-defence systems to resist host defence through anti-restriction strategies [77]. Over 200 viral genes related to the RM systems of types I, II and III are present in marine biofilms and endow biofilm viruses with the capability to modify viral DNA. Thus, the recognition of host restriction enzymes can be prevented and viral genome cleavage can be avoided.

However, prokaryotes can employ multiple defence lines for fighting viruses [85]. Our study on archaeal and bacterial genomes showed that hundreds of thousands of genes are related to 17 defence systems in marine biofilms. The expression of these systems in microbial communities in HK-2022 biofilms indicates that multiple systems exert a synergistic effect that efficiently removes invading viruses [86]. For instance, when the phages biofilm1_vRhyme_bin_46 and biofilm1_vRhyme_bin_52 inject their genetic materials into the bacterial cells of biofilm1_bin.9 in the family of *Alteromonadaceae*, the activity of the CRISPR–Cas system encoding Cas1, Cas2 and Cas9 (Table S17) for cleaving invading DNA and incorporating viral sequences as spacers for memory was observed. Additionally, TA systems, including the type II TA encoding the ParE toxins and ParD antitoxins [87] in biofilm1_bin.9, can employ toxins to inhibit cell proliferation and inhibit viral replication. Other systems (e.g. HACHIMAN, Thoeris and Shedu) with unknown mechanisms might play a complementary role for *Alteromonadaceae* biofilm1_bin.9 in the presence of invading phages. Along with known mechanisms, such as quorum sensing under phage predation, abundant defence systems contribute to prokaryotic genotypic evolution and facilitate coexistence with viruses in marine biofilms.

Compared with defence systems in seawater microbiomes, biofilm prokaryotes displayed higher abundance and difference in composition, consistent with the observation that biofilm bacteria and archaea are unique communities [11]. Abundant defence mechanisms can confer resistance to viral infection on prokaryotes, as evidenced by a study revealing that enriched defence systems reduce the infectivity of the phage strains of *Pseudomonas aeruginosa* [88]. Given the high level of resistance, we hypothesised that marine biofilms are habitats for the blooming of anti-viral defence strategists. Seawater is conducive to the rapid growth of competition strategists but it restricts the development of defence strategists. A switch from free-living to being sessile will make defence strategists dominant in microbial communities. This switch will also benefit the stability of microbial communities in seawater under viral predation when prokaryotes go back to a free-living style via detachment. The abundance and composition of defence genes are linked to substrate surfaces. Cell culture Petri dishes made of polystyrene support the colonisation of defence-preferring microorganisms, whereas zinc panels inhibit the blooming of defence systems in biofilm communities. Zinc can cause biofilm biomass reduction in marine habitats [89] and is unfavourable to the biofilm development of certain species, such as *Actinobacillus pleuropneumoniae* [90]. Phage biocontrol has been applied to treat environmentally detrimental biofilms in the food industry [91] and has been tested in water systems [92, 93]. Phages facilitate the control of biofilms, but surface substrates to which biofilms attach should not be overlooked. Biofilms on specific materials may enrich defence strategists and counter the effect of phage biocontrol. Finally, enriched defence systems can provide a catalogue of defence-related genes in marine environments, and further exploration would expand the defence systems database and contribute to immune research.

### Supplementary information

Supplementary Results and Figures

Supplementary Tables

## Data Availability

The data of HK-2022 biofilms that support the findings of this study are deposited into the NCBI database under the BioProject ID PRJNA983852.
