# Supplementary material for: Genomic and transcriptomic insights into complex virus–prokaryote interactions in marine biofilms
Source: ISME J. 2023 Oct 24;17(12):2303–12. doi: 10.1038/s41396-023-01546-2 (PMC10689801; doi:10.1038/s41396-023-01546-2)
Supplement: Supplementary file 1 — Supplementary Results and Figures [file 41396_2023_1546_MOESM1_ESM.docx]

**Supplementary Results**

**1. Complete anti-viral defence systems in biofilm prokaryotic bins paired with viruses**

The type II Zorya system in *Oceanisphaera* SRR6869393.1_bin.16 and Shedu system in the *Pleurocapsa* SRR6869023.1_bin.26 of *Cyanobacteria* were exclusively composed of the single the genes *zorE* and *sduA*, respectively. The Hachiman system contained a pair of genes, *hamA* and *hamB*, in the scaffold NODE_2158 of SRR6854716.1_bin.13 that was affiliated with *Francisellaceae*. The system of Lamassu was also composed of a couple of genes, *lmuA* and *lmuB*, in *Tateyamaria* SRR6869054.1_bin.19. The Gabija system in the SRR6854573.1_bin.4 in the order of *Opitutales* displayed two gene components (*gajA* and *gajB*) near to each other. The Wadjet system in SRR6854591.1_bin.2 in *Gammaproteobacteria* contained the genes *jetA*, *jetB*, *jetC* and *jetD*. The Septu system was composed of genes encoding *PtuA* and *PtuB* in cyanobacterium SRR6854590.1_bin.4 in the family *Elainellaceae*. Type II TA systems had multiple types, such as TA gene pairs for BrnA (antitoxin) and BrnT (toxin) in SRR6854573.1_bin.1 in the family of *Microcoleaceae* and ParD antitoxin and ParE toxin in *Vibrio* SRR6854588.1_bin.6. In *Vibrio* SRR6854588.1_bin.3, the foreign DNA-targeting RM system encompassed a pair of genes for a restriction enzyme (cleaving specific DNA sites) and methyltransferase (modifying nucleotides by methylation). As for the adaptive CRISPR–Cas systems, type II-C CRISPR–Cas system harboured in the SRR6854716.1_bin.15 of *Alphaproteobacteria* displayed interference and adaptation genes encoding Cas2, Cas1 and HNH endonuclease. Additionally, the CRISPR spacers near the gene for Cas2 exactly matched the sequence of NODE_980 of *Uroviricota* viruses.

**2. Exprssion of viral hallmark genes**

In the genomes of viruses paired with prokaryotic hosts, viral genes with read or transcript support harboured a variety of hallmark genes. These hallmarks encompassed the capsid gene in *Acidimicrobiales* phage biofilm1_vRhyme_bin_22, the gene encoding the portal protein in *Silicimonas* phage biofilm3_vRhyme_bin_51, the terminase large subunit gene in *Methylophilaceae* phage biofilm2_vRhyme_bin_55, the gene for phage virion morphogenesis in *Alteromonadaceae* phage biofilm1_vRhyme_bin_52, the genes encoding phage integrase family in *Silicimonas* phage (biofilm1_NODE_463_length_15656_cov_4.739632) and the genes encoding tail tube proteins, tail sheath proteins and tail assembly chaperone proteins in *Alteromonadaceae* phage biofilm2_vRhyme_bin_54 (with all trinity support). The expression of the viral hallmark genes demonstrated that viruses play an active role in targeting prokaryotic hosts in biofilms.

**Supplementary Figures**

**Figure S1.** The relative abundance of prokaryotic bins paired/unpaired with viruses in marine biofilms. The vertical axis is the value of the relative abundance of prokaryotes. Prokaryotic genome bins paired/unpaired with identified viruses and sequenced metagenomic reads were input into Bowtie2 version 2.3.4 [1] and SAMtools version 1.6 [2] to calculate average sequencing depth. The read coverage of prokaryotes that were paired or unpaired with identified viruses in a single biofilm was summed up separately.


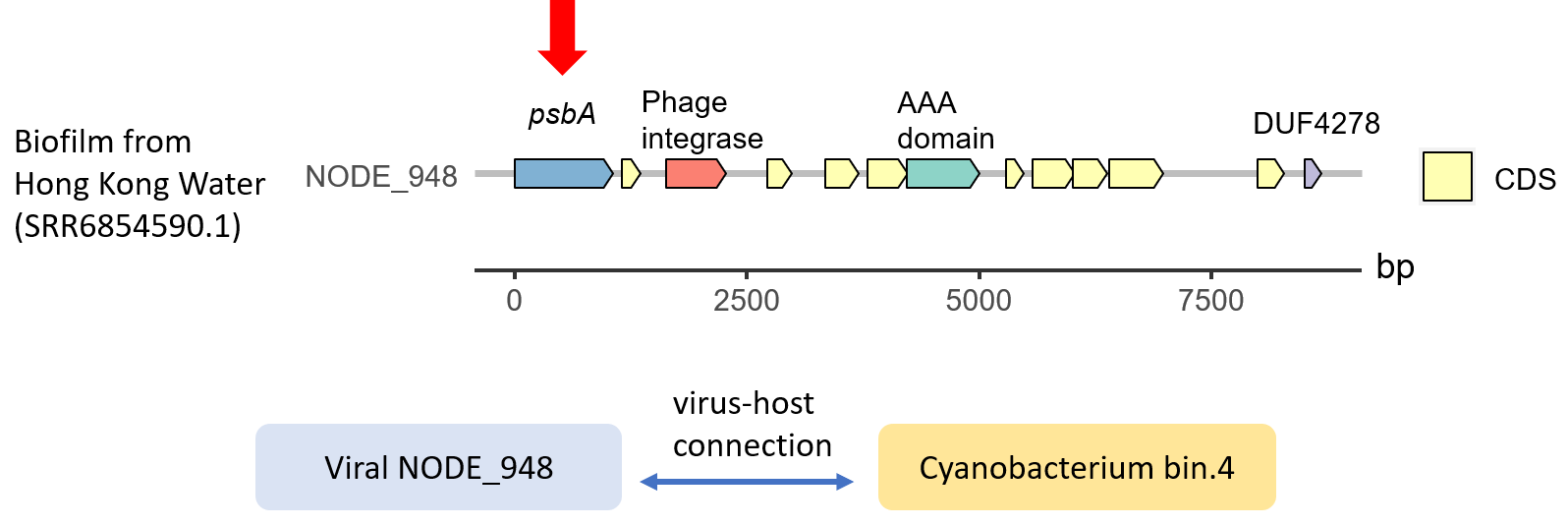


**Figure S2.** Gene *psbA* in the viral scaffold NODE_948. By using phage-host prediction, it was determined that the viral NODE_948 was associated with the Cyanobacterium bin.4.


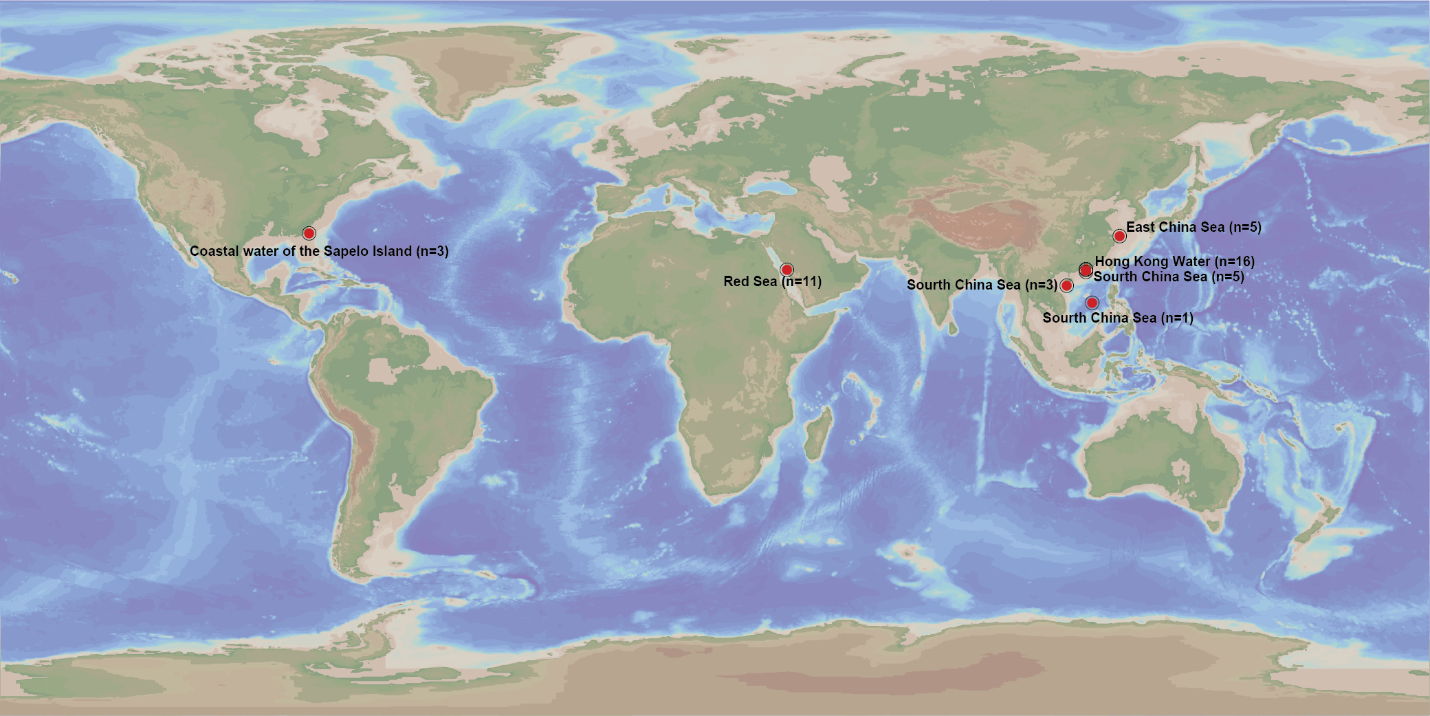


**Figure S3.** Spatial distribution of *nrdA* in global marine biofilms. The number of biofilms containing the *nrdA* gene in each location was indicated within brackets.


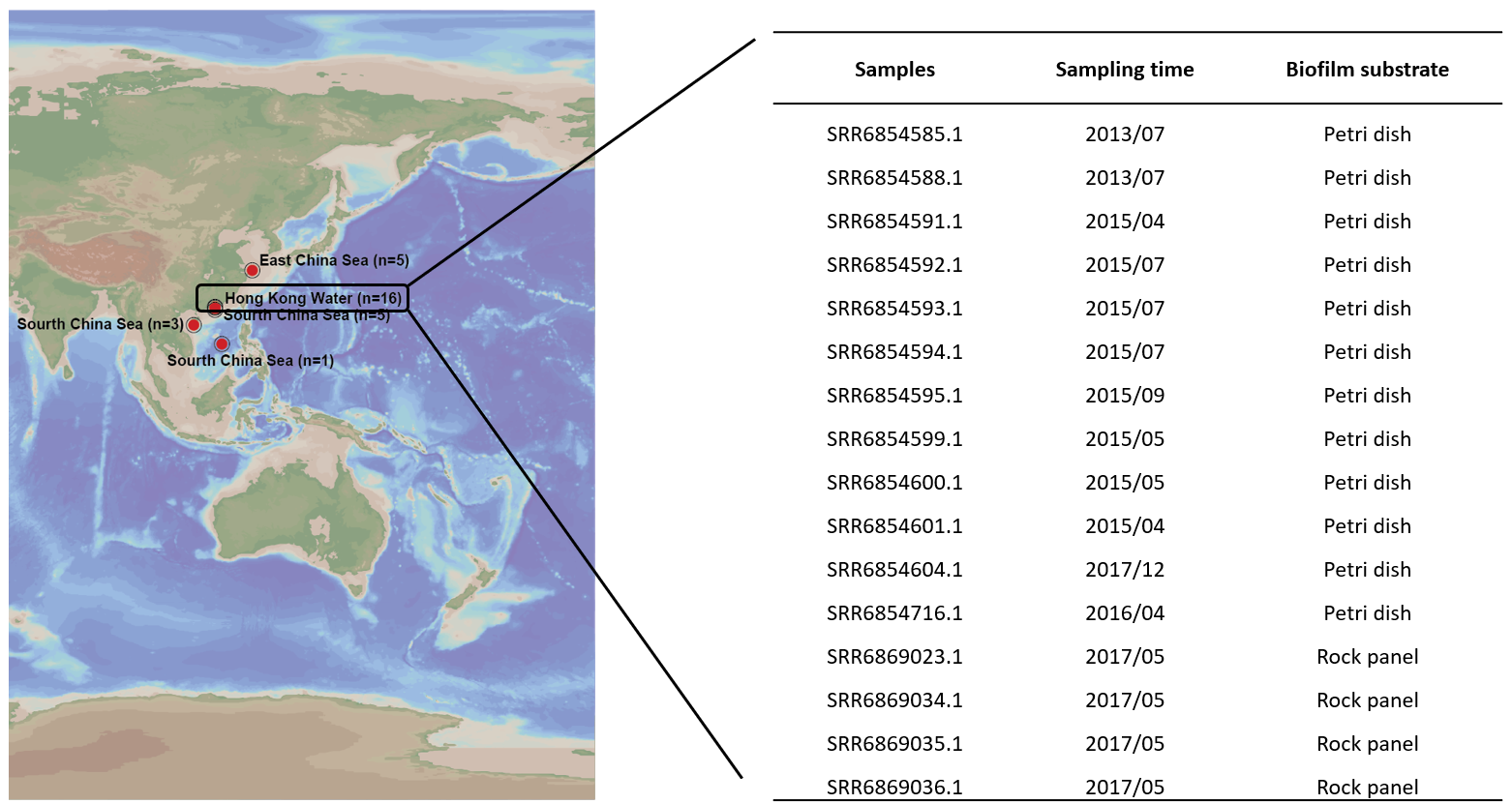


**Figure S4.** Temporal distribution of *nrdA* in marine biofilms developed at Hong Kong Waters from 2013 to 2017.

**References**

1. Langmead B, Salzberg SL. Fast gapped-read alignment with Bowtie 2. *Nat. Methods*. 2012; **9**: 357-359.

2. Li H, Handsaker B, Wysoker A, Fennell T, Ruan J, Homer N *et al*. The sequence alignment/map format and SAMtools. *Bioinformatics*. 2009; **25**: 2078-2079.
